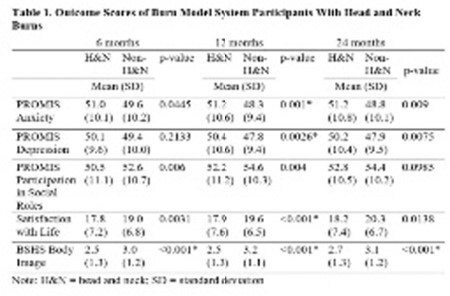# 564 Adults with Head and Neck Burns Have Worse Long-Term Psychosocial Outcomes

**DOI:** 10.1093/jbcr/irae036.198

**Published:** 2024-04-17

**Authors:** Deborah Choe, Kara McMullen, Barclay T Stewart, Karen J Kowalske, Jeffrey C Schneider, Colleen M Ryan, Lewis E Kazis, Caitlin M Orton, Haig A Yenikomshian

**Affiliations:** Keck School of Medicine of USC, Los Angeles, CA; University of Washington, Seattle, WA; UT Southwestern Medical Center, Dallas, Dallas, TX; Spaulding Rehabilitation Hospital/Harvard Medical School, Boston, MA; Massachusetts General Hospital/Shriners Children's, Boston, MA; Department of Health Law, Policy and Management, Boston University School of Public Health, Boston, MA; UW Medicine Regional Burn Center, Harborview Medical Center, Seattle, WA; University of Southern California, Los Angeles, CA; Keck School of Medicine of USC, Los Angeles, CA; University of Washington, Seattle, WA; UT Southwestern Medical Center, Dallas, Dallas, TX; Spaulding Rehabilitation Hospital/Harvard Medical School, Boston, MA; Massachusetts General Hospital/Shriners Children's, Boston, MA; Department of Health Law, Policy and Management, Boston University School of Public Health, Boston, MA; UW Medicine Regional Burn Center, Harborview Medical Center, Seattle, WA; University of Southern California, Los Angeles, CA; Keck School of Medicine of USC, Los Angeles, CA; University of Washington, Seattle, WA; UT Southwestern Medical Center, Dallas, Dallas, TX; Spaulding Rehabilitation Hospital/Harvard Medical School, Boston, MA; Massachusetts General Hospital/Shriners Children's, Boston, MA; Department of Health Law, Policy and Management, Boston University School of Public Health, Boston, MA; UW Medicine Regional Burn Center, Harborview Medical Center, Seattle, WA; University of Southern California, Los Angeles, CA; Keck School of Medicine of USC, Los Angeles, CA; University of Washington, Seattle, WA; UT Southwestern Medical Center, Dallas, Dallas, TX; Spaulding Rehabilitation Hospital/Harvard Medical School, Boston, MA; Massachusetts General Hospital/Shriners Children's, Boston, MA; Department of Health Law, Policy and Management, Boston University School of Public Health, Boston, MA; UW Medicine Regional Burn Center, Harborview Medical Center, Seattle, WA; University of Southern California, Los Angeles, CA; Keck School of Medicine of USC, Los Angeles, CA; University of Washington, Seattle, WA; UT Southwestern Medical Center, Dallas, Dallas, TX; Spaulding Rehabilitation Hospital/Harvard Medical School, Boston, MA; Massachusetts General Hospital/Shriners Children's, Boston, MA; Department of Health Law, Policy and Management, Boston University School of Public Health, Boston, MA; UW Medicine Regional Burn Center, Harborview Medical Center, Seattle, WA; University of Southern California, Los Angeles, CA; Keck School of Medicine of USC, Los Angeles, CA; University of Washington, Seattle, WA; UT Southwestern Medical Center, Dallas, Dallas, TX; Spaulding Rehabilitation Hospital/Harvard Medical School, Boston, MA; Massachusetts General Hospital/Shriners Children's, Boston, MA; Department of Health Law, Policy and Management, Boston University School of Public Health, Boston, MA; UW Medicine Regional Burn Center, Harborview Medical Center, Seattle, WA; University of Southern California, Los Angeles, CA; Keck School of Medicine of USC, Los Angeles, CA; University of Washington, Seattle, WA; UT Southwestern Medical Center, Dallas, Dallas, TX; Spaulding Rehabilitation Hospital/Harvard Medical School, Boston, MA; Massachusetts General Hospital/Shriners Children's, Boston, MA; Department of Health Law, Policy and Management, Boston University School of Public Health, Boston, MA; UW Medicine Regional Burn Center, Harborview Medical Center, Seattle, WA; University of Southern California, Los Angeles, CA; Keck School of Medicine of USC, Los Angeles, CA; University of Washington, Seattle, WA; UT Southwestern Medical Center, Dallas, Dallas, TX; Spaulding Rehabilitation Hospital/Harvard Medical School, Boston, MA; Massachusetts General Hospital/Shriners Children's, Boston, MA; Department of Health Law, Policy and Management, Boston University School of Public Health, Boston, MA; UW Medicine Regional Burn Center, Harborview Medical Center, Seattle, WA; University of Southern California, Los Angeles, CA; Keck School of Medicine of USC, Los Angeles, CA; University of Washington, Seattle, WA; UT Southwestern Medical Center, Dallas, Dallas, TX; Spaulding Rehabilitation Hospital/Harvard Medical School, Boston, MA; Massachusetts General Hospital/Shriners Children's, Boston, MA; Department of Health Law, Policy and Management, Boston University School of Public Health, Boston, MA; UW Medicine Regional Burn Center, Harborview Medical Center, Seattle, WA; University of Southern California, Los Angeles, CA

## Abstract

**Introduction:**

People living with burns of the head and neck (H&N) often experience serious physical (e.g., microstomia, ectropion) and psychological sequelae (e.g., adjustment and mood disorders). We aimed to compare long-term outcomes in key psychosocial domains between adults with and without H&N burns.

**Methods:**

Adults with burn injuries after 2014 enrolled in the Burn Model System national longitudinal, multicenter database were included. Survey responses using the PROMIS-29 (Anxiety, Depression, and Participation in Social Roles), Satisfaction with Life Scale (SWL), and Burn Specific Health Scale (BSHS) at 6-, 12-, and 24 months post-injury were analyzed. Differences between participants with and without H&N burns were examined with non-parametric tests.

**Results:**

This study included 1,247 participants: 579 had H&N burns and 668 had non-H&N burns. Compared to those with non-H&N burns, participants with H&N burns had significantly larger burn size (22.3 ±18.2 vs. 8.6 ±11.0 % total body surface area (TBSA), p< 0.001) and longer hospital stays (32.6 ±38.3 vs. 16.9 ±16.5 days, p< 0.001). At 12 months, participants with H&N burns reported significantly worse anxiety (51.2 ±10.6 vs. 48.3 ±9.4, p=0.001), depression (50.4 ±10.6 vs. 47.8 ±9.4, p=0.0026), and SWL (17.9 ±7.6 vs. 19.6 ±6.5, p< 0.001). Participants with H&N burns also reported significantly worse body image at 6 months (2.5 ±1.3 vs. 3.0 ±1.2, p< 0.001), 12 months (2.5 ±1.3 vs. 3.2 ±1.1, p< 0.001), and 24 months (2.7 ±1.3 vs. 3.1 ±1.2, p< 0.001).

**Conclusions:**

Participants with H&N burns reported significantly worse outcomes in several psychosocial domains, including anxiety, depression, life satisfaction, and body image. These findings and evidence from early psychological intervention suggest that the presence of H&N burns is a potentially useful screening tool to identify an adult population who might benefit from early evaluation and treatment for psychological stress, coping and adjustment (e.g., cognitive processing therapy).

**Applicability of Research to Practice:**

In addition to future studies, this study may be used when considering recommendations for psychotherapy or counseling services available to people living with H&N burns.